# Socioeconomic and Other Demographic Disparities Predicting Survival among Head and Neck Cancer Patients

**DOI:** 10.1371/journal.pone.0149886

**Published:** 2016-03-01

**Authors:** Seung Hee Choi, Jeffrey E. Terrell, Karen E. Fowler, Scott A. McLean, Tamer Ghanem, Gregory T. Wolf, Carol R. Bradford, Jeremy Taylor, Sonia A. Duffy

**Affiliations:** 1 College of Nursing, Michigan State University, East Lansing, MI, United States of America; 2 University of Michigan Health System, Ann Arbor, MI, United States of America; 3 Center for Clinical Management Research, VA Ann Arbor Healthcare System, Ann Arbor, MI, United States of America; 4 Henry Ford Hospital, Detroit, MI, United States of America; 5 Department of Computational Medicine & Bioinformatics, University of Michigan, Ann Arbor, MI, United States of America; 6 College of Nursing, Ohio State University, Columbus, OH, United States of America; University of Cincinnati College of Medicine, UNITED STATES

## Abstract

**Background:**

The Institute of Medicine (IOM) report, “Unequal Treatment,” which defines disparities as racially based, indicates that disparities in cancer diagnosis and treatment are less clear. While a number of studies have acknowledged cancer disparities, they have limitations of retrospective nature, small sample sizes, inability to control for covariates, and measurement errors.

**Objective:**

The purpose of this study was to examine disparities as predictors of survival among newly diagnosed head and neck cancer patients recruited from 3 hospitals in Michigan, USA, while controlling for a number of covariates (health behaviors, medical comorbidities, and treatment modality).

**Methods:**

Longitudinal data were collected from newly diagnosed head and neck cancer patients (N = 634). The independent variables were median household income, education, race, age, sex, and marital status. The outcome variables were overall, cancer-specific, and disease-free survival censored at 5 years. Kaplan-Meier curves and univariate and multivariate Cox proportional hazards models were performed to examine demographic disparities in relation to survival.

**Results:**

Five-year overall, cancer-specific, and disease-free survival were 65.4% (407/622), 76.4% (487/622), and 67.0% (427/622), respectively. Lower income (HR, 1.5; 95% CI, 1.1–2.0 for overall survival; HR, 1.4; 95% CI, 1.0–1.9 for cancer-specific survival), high school education or less (HR, 1.4; 95% CI, 1.1–1.9 for overall survival; HR, 1.4; 95% CI, 1.1–1.9 for cancer-specific survival), and older age in decades (HR, 1.4; 95% CI, 1.2–1.7 for overall survival; HR, 1.2; 95% CI, 1.1–1.4 for cancer-specific survival) decreased both overall and disease-free survival rates. A high school education or less (HR, 1.4; 95% CI, 1.0–2.1) and advanced age (HR, 1.3; 95% CI, 1.1–1.6) were significant independent predictors of poor cancer-specific survival.

**Conclusion:**

Low income, low education, and advanced age predicted poor survival while controlling for a number of covariates (health behaviors, medical comorbidities, and treatment modality). Recommendations from the Institute of Medicine’s Report to reduce disparities need to be implemented in treating head and neck cancer patients.

## Introduction

Cancer disparities are endemic in the United States healthcare system and in many other industrialized nations. Disparities may be characterized by socioeconomic status (SES), including income, education, ethnicity/race, age, sex, and marital status, as well as other factors such as insurance, disability, geographic location, or sexual orientation.[1–5] The US Department of Health and Human Services’ Healthy People 2020 initiative has identified the elimination of health disparities as one of its overarching goals.[6] However, the Institute of Medicine (IOM) report, “Unequal Treatment,” which defines disparities as racially based, indicates that disparities in cancer diagnosis and treatment are less clear than disparities in other diagnoses such as cardiac care.[7] Nonetheless, a number of studies have been conducted that have begun to address disparities among cancer patients.

Several studies have noted racial differences in both survival and/or recurrence among head and neck cancer patients.[3, 5, 8–19] Other studies indicate that minorities tend to present at a later cancer stage at diagnosis and less likely to have insurance, which suggests that equal access to care may eliminate racial disparities.[10, 12, 15, 20–22] Some research including head and neck cancer patients,[9, 12, 23] demonstrates that when controlling for SES and behavioral factors (e.g., smoking), racial disparities are diminished or no longer present.[9, 12, 21, 23–25]

Among cancer patients, age has been shown to predict survival in most studies, which can partially be explained by comorbidities and/or differential treatment given to older persons,[5, 13, 26–28] yet other studies have shown no age differences in survival in head and neck cancer patients.[10, 29] While women with head and neck cancer have been demonstrated to live longer than men,[5, 17–19, 30] other studies have indicated that sex was not predictive of survival or recurrence among head and neck cancer patients.[5, 8, 31–33] A relationship between marital status and survival and recurrence has also been reported among cancer patients.[17, 18, 31, 34, 35]

Limitations on the studies of disparities among head and neck cancer patients include their retrospective nature,[8, 10, 17] inability to control for covariates,[14, 19, 23, 32, 36] measurement errors (such as using postal address as a proxy for SES[5, 10] or using a county-level variables as a proxy of SES[17]), and small sample sizes.[31] Determining the nature and extent of disparities is important in identifying interventions to reduce disparities. Using data from a large longitudinal study, the specific aim of this study was to examine disparities as predictors of 5-year overall, cancer-specific, and disease-free survival among newly diagnosed head and neck cancer patients.

## Materials and Methods

### Study Design

This was a prospective observational longitudinal study of patients enrolled in the University of Michigan Head and Neck Cancer Specialized Program of Research Excellence (SPORE). The independent variables were income, education, ethnicity/race, age, sex, and marital status. Covariates were smoking,[5, 37] problem drinking,[5, 38] body mass index (BMI),[39, 40] depression,[4, 41] cancer site,[2, 5, 9, 12] cancer stage,[5, 17, 42] comorbidities,[4, 5] and treatment.[5, 42] The outcome variables were overall, cancer-specific, and disease-free survival censored at 5 years post-diagnosis or April 1, 2009, whichever came first. Human subjects approval was received from the Medical School Institutional Review Board (IRBMED) at the University of Michigan, the VA Ann Arbor Healthcare System, and Henry Ford Hospital. Recruitment was conducted from January 2003 to November 2008.

### Study Population

Newly diagnosed patients with squamous cell carcinoma of the head and neck were recruited to participate in this study. To help ensure a diverse patient population of minorities and those of lower socioeconomic status, in addition to recruiting patients from the University of Michigan, patients were also recruited from the Veterans Affairs (VA) Ann Arbor Healthcare System, and Henry Ford Hospital in Detroit. Exclusion criteria were those: 1) less than 18 years of age; 2) pregnant; 3) non-English speaking; 4) mentally unstable; 5) with non-upper aerodigestive tract cancers (such as thyroid or skin cancer); 6) with a historical diagnosis and treatment for head and neck cancer; or 7) in stage 0 at diagnosis. Out of 1185 patients approached, 934 consented to participate, yielding a response rate of 79%. Of those consented, 796 met all eligibility requirements for this analysis. Survival curves and additional analyses included only subjects with no missing survey data, leaving a sample size of 622 (78% of eligible patients). Those with missing data were significantly more likely to be black, older age, unmarried, current smokers, and have more comorbidities, but did not have problem drinking and not receive radiation or chemotherapy (Table 1).

**Table 1:** Comparison between those included and those excluded in the analysis.

	Univariate models		
Parameter	Included	Excluded	*p* value
	(n = 622)	(n = 174)	
**Ethnicity/race**			< .001
White	548 (79.8)	139 (20.2)	
Black	41 (60.3)	27 (39.7)	
Hispanic, other (Native American)	33 (89.2)	4 (10.8)	
**Age** (in decades)	58.4 (10.8)	61.6 (12.5)	.003
**Marital status**			.037
** **Married	374 (81.3)	86 (18.7)	
** **Separated/Widowed/Divorced	248 (75.2)	82 (24.8)	
**Smoking status**^**b**^			.009
** **Current smoker	155 (71.4)	62 (28.6)	
** **Former smoker	358 (81.7)	80 (18.3	
** **Never	109 (80.2)	27 (19.8)	
**Problem drinking**^**c**^			.004
** **Yes	160 (88.9)	20 (11.1)	
** **No	462 (79.3)	121 (20.7)	
**ACE-27 comorbidity score**			.008
** **None	179 (82.5)	38 (17.5)	
** **Mild	237 (81.4)	54 (18.6)	
** **Moderate	138 (75.8)	44 (24.2)	
** **Severe	68 (67.3)	33 (32.7)	
**Radiation treatment**			.008
** **Received	541 (82.3)	116 (17.7)	
** **Not Received	81 (71.7)	32 (28.3)	
**Chemotherapy treatment**			.001
** **Received	427 (84.2)	80 (15.8)	
** **Not Received	195 (74.4)	67 (25.6)	

Median income, education, sex, BMI, depression, cancer site, stage, surgery, and hospital site were not significantly different between those included in the analysis and those excluded in the analysis.

### Procedure

Research assistants recruited patients to the study in the waiting rooms of otolaryngology clinics. Written informed consent was obtained, and patients completed written surveys on demographics and health behaviors. A medical record review was conducted for each study participant. Subjects enrolled in the study were then resurveyed every 3 months for 2 years, and yearly thereafter.

#### Independent Variables

Median household income for the census tract of each subject was found using American Fact Finder data for the 2000 US Census from the www.census.gov website. Low income was defined as the lowest quartile of incomes (<$35,000). Standard questions on demographics were collected from the patient surveys, including education, ethnicity/race, age, sex, and marital status. Ethnicity/race was measured using two separate questions about Hispanic/Latino origin and race. Due to sample size limitations, ethnicity/race was classified as white, black, or Hispanic/other (e.g., Native American).

#### Covariates

Covariates were determined based on the current literature and clinical judgement and were controlled by constructing multivariate Cox proportional hazard models. Smoking status was characterized as current, former, or never smoking tobacco products (including cigarettes, cigars, and pipe tobacco) at diagnosis. The previously validated 10-item instrument, Alcohol Use Disorders Identification Test (AUDIT),[43] was used to measure alcohol use; the scores ranged from 0 to 40 with a score of 8 or more indicating problem drinking.[44] BMI (weight in kilograms divided by the square of height in meters) was calculated based on self-reported height (without shoes) and weight. Depressive symptoms were measured using the 5-item Geriatric Depression Scale–Short Form (GDS-SF), with a score of 4 or more indicating probable depression.[45] Cancer sites were classified into four groups: a) oral cavity, b) oropharynx, c) larynx, and d) other (hypopharynx, nasopharynx, sinus, and others). Cancer stage (I–IV) and TNM classification were measured using the American Joint Committee on Cancer (AJCC) staging classification system.[46, 47] Comorbidities were measured using the Adult Comorbidity Evaluation-27 (ACE-27) and classified as none, mild, moderate, or severe.[48, 49] Type of curative treatment received (surgery, radiation, and/or chemotherapy [yes/no]) was recorded by yearly chart audit or patient self-report when treated at an outside facility.

#### Outcome Variable

The three main outcome variables were overall, cancer-specific, and disease-free survival. Survival was defined by the number of days from the date of initial diagnosis until the date of death from either all-cause (overall survival), cancer-related causes (cancer-specific survival), or the date of first recurrence (disease-free survival). Patients were contacted every 3 months to keep track of survival (dead or alive) and recurrence status (recurrence or recurrence-free) for the first 2 years after diagnosis and then yearly thereafter. If patients were lost to follow-up, the Social Security Administration Death Master File (DMF) was used to determine if and when they had died. Patients lost to follow-up and not found on the DMF were assumed alive. Subjects who were alive or free of recurrence at 5 years post-diagnosis were censored on April 1, 2009.

### Statistical Analysis

Means and frequency distributions were examined for all variables. Associations between independent variables were conducted using chi-square for categorical variables, t-tests, and analysis of variance for continuous variables. All independent variables and covariates were treated as categorical variables except age and BMI. Kaplan-Meier plots and the log-rank test were used to compare the independent variables and survival. Univariate and multivariate Cox proportional hazards models were used to study the relationship between the independent variables, covariates, and dependent variables. Since hospital site was significantly correlated with income, education, race, and marital status, it was removed from the multivariate models. TNM classification was not included in the multivariate models because there were so few M1 (1.1%, n = 7) and TN classification were highly collinear with cancer stage; the Variance Inflation Factor (VIF) for stage increased from 3.8 to 7.0 when TN classification added to the model. The VIF values ranged from 1.0 through 3.7 indicating no significant multicollinearity among variables in the multivariate models.[50] Values for *p* < .05 were reported.

## Results

### Description of the Sample

The patient characteristics are described in Table 2 (*N* = 622). The median household income was $43,996. There were nearly equal numbers of patients who had attended some college or more as those with a high school education or less. The mean age was 58 years. Most of the respondents were non-Hispanic whites (*n* = 548, 88.1%). Just over three-quarters were male (*n* = 491, 78.9%), and more than half were married (*n* = 374, 60.1%).

**Table 2:** Pretreatment patient characteristics of newly diagnosed head and neck cancer patients. (N = 622).

Parameter	Mean (SD)/median	Range
**Median follow-up time in days**	1,445.5 days	19–1,826 days
**Median household income**	$43,996	$11,232-$137,720
**Mean age in years**	58.4 years (10.8)	21–92 years
**Mean BMI**	26.8 (5.7)	15.2–64.6
	No. of patients	Percentage
**Educational level**		
High school or less	297	47.7
Some college or more	325	52.3
**Ethnicity/race**		
Non-Hispanic white	548	88.1
Black	41	6.6
Hispanic, other (Native American)	33	5.3
**Sex**		
Male	491	78.9
Female	131	21.1
**Marital status**		
Married	374	60.1
Not married	248	39.9
**Smoking status**^a^		
Current smoking	155	24.9
Former smoking	358	57.6
Never smoking	109	17.5
**Problem drinking**^b^	160	25.7
**Depression**^c^	323	51.9
**Cancer site**		
Oral cavity	136	21.9
Oropharynx	249	40.0
Larynx	140	22.5
Hypopharynx	34	5.5
Nasopharynx	8	1.3
Sinus	44	7.1
Other	5511	1.8
**Cancer stage**		
I	65	10.5
II	60	9.7
III	93	15.0
IV	404	65.0
**TNM Classification**		
T Stage		
TX	46	7.4
T1	121	19.5
T2	158	25.4
T3	111	17.8
T4	186	29.9
N Stage		
NX	1	0.2
N0	213	34.2
N1	82	13.2
N2	269	43.3
N3	57	9.2
M Stage		
MX	29	4.7
M0	586	94.2
M1	7	1.1
**ACE-27 comorbidity**		
None	179	28.8
Mild	237	38.1
Moderate	138	22.2
Severe	68	10.9
**Treatment**		
Radiation + Chemotherapy + Surgery	167	26.9
Radiation + Chemotherapy	253	40.7
Radiation + Surgery	72	11.6
Chemotherapy + Surgery	2	0.3
Radiation Only	49	7.9
Chemotherapy Only	5	0.8
Surgery Only	68	10.9
No Treatment	6	1.0
**Hospital site**		
University of Michigan	479	77.0
Veterans Affairs Ann Arbor	63	10.1
Henry Ford Hospital	80	12.9

^a^ Includes cigarettes, cigars, and pipe tobacco.

^b^ Alcohol Use Disorders Identification Test (AUDIT) ≥8.

^c^ Geriatric Depressive Scale Short Form ≥4.

The majority of patients were current (*n* = 155, 24.9%) or former (*n* = 358, 57.5%) smokers. About one-quarter screened positive for problem drinking (*n* = 160, 25.7%), and half screened positive for significant depressive symptoms (*n* = 323, 51.9%). The mean BMI was 26.8 kg/m^2^ with more than half of the patients being either overweight (*n* = 233, 37.5%) or obese (*n* = 141, 22.7%). More than one-third of the sample had oropharyngeal cancer (*n* = 249, 40.0%), followed by laryngeal cancer (*n* = 140, 22.5%) and oral cavity cancer (*n* = 136, 21.9%). Almost two-thirds of the patients presented with stage IV disease (*n* = 404, 65.0%) and had none (*n* = 179, 28.8%) to mild (*n* = 237, 38.1%) comorbidities at diagnosis. The majority of patients received radiation (87.0%), chemotherapy (68.7%), and surgery (49.7%) and received a combination of treatments (e.g., radiation and chemotherapy, radiation and chemotherapy and surgery). The 5-year overall survival rate was 65.4% (*n* = 407/622), cancer-specific survival rate was 76.4% (*n* = 475/622), and disease-free survival rate was 67.0% (*n* = 417/622).

### Associations Among Independent Variables

While low income and education were not associated with each other (*p* = .309), both were associated with black race (*p* < .0001 and *p* = .002, respectively), older age (*p* = .038 and *p* = .003, respectively), being unmarried (*p* = .002 and *p* = .0001, respectively), current smoking (*p* = .039 and *p* = .010, respectively), and cancer site (*p* = .010 and *p* < .0001, respectively), with higher education and higher income being more likely to have cancers of the oropharynx. In addition, lower educational levels were associated with being female (*p* = .024), problem drinking (*p* = .002), and higher levels of comorbidities (*p* = .001). Blacks were more likely to be unmarried (*p* = .018), have low BMI (*p* = .008), and not be receiving chemotherapy (*p* = .002).

Older patients tended not to have problem drinking (*p* = .0003), but have low BMI (*p* = .015), less depression (*p* = .004), and more comorbidities (*p* < .0001). While older persons had more cancers of the larynx, younger persons had more cancers of the oral cavity and oropharynx (*p* = .0003). Younger persons were more likely to receive surgery (*p* = .008) and chemotherapy (*p* = .0008). Female patients tended to be unmarried (*p* = .0002) and have, cancers of the oral cavity (*p* < .0001), and surgery treatment (*p* = .051) but not to have either problem drinking (*p* = .0002) or radiation (*p* = .009) or chemotherapy (*p* = .003).

### Univariate and Multivariate Survival Analyses

**Fig 1:**
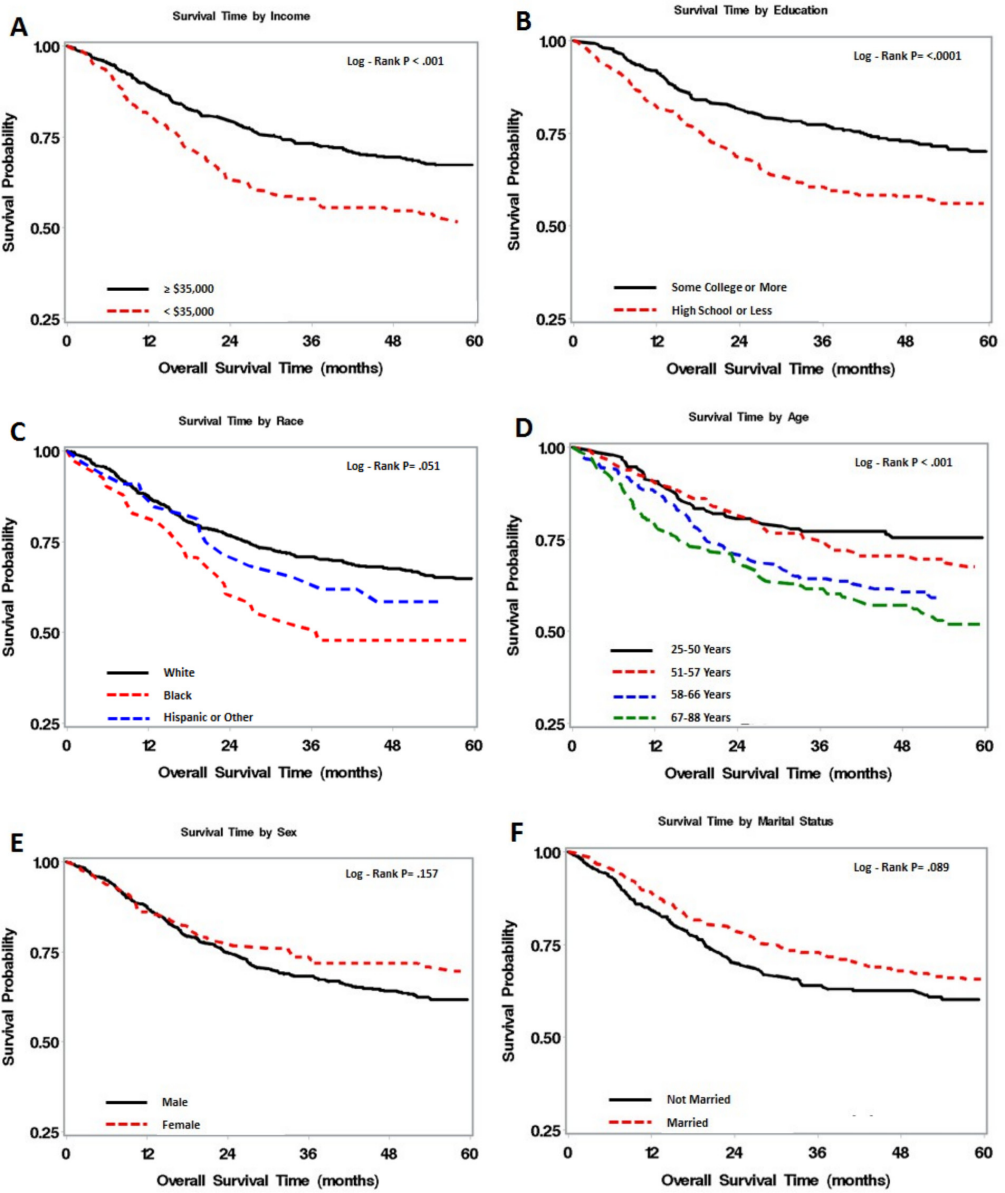
Kaplan-Meier survival curves for the independent variables.

Fig 1 shows the Kaplan-Meier survival curves for the independent variables of income, educational level, ethnicity/race, age, sex, and marital status. Those in the lowest income quartile had the worst survival compared to all others (*p* < .001). Patients with a high school education or less had poorer survival compared to those with some college or more education (*p* < .0001). Blacks had worse survival compared to either white or Hispanic/other race groups (*p* = .051). The Kaplan-Meier curve revealed a significant association with survival for age, with those in the oldest age quartile having the worst survival (*p* < .001). Survival rates were similar for males and females for the first 2 years, but then diverged with a trend for men having worse survival than women (*p* = .157). Those who were not married trended toward poorer survival compared to those who were, albeit not significant (*p* = .089).

Table 3 shows the univariate and multivariate hazard ratios of overall survival for each of the independent and covariates. Univariate analysis revealed that income, education, ethnicity/race, age, smoking status, problem drinking, BMI, cancer sites, cancer stage, and comorbidities were all significantly associated with overall survival. Not being married trended toward poorer survival in the univariate models, but along with sex, depressive symptoms, and treatment (surgery, radiation, and chemotherapy),was not significantly associated.

**Table 3:** Univariate and multivariate Cox proportional hazards model for 5-year overall survival. (N = 634 [216 events, 418 censored]).

	Univariate models			Multivariate models		
Parameter	Hazard ratio	95% CI	*p* value	Hazard ratio	95% CI	*p* value
**Low income**^a^	1.70	1.28–2.26	< .001*	1. 48	1.09–2.01	.013*
**High school education or less**	1.72	1.31–2.26	< .001*	1. 41	1.05–1.88	.022*
**Ethnicity/Race** (vs white)						
Black	1.77	1.14–2.76	.011*	1. 33	0.81–2.17	.262
Hispanic, other (Native American)	1.01	0.47–2.14	.985	0. 92	0.42–2.03	.836
**Age** (in decades)	1.37	1.21–1.55	< .001*	1. 43	1.23–1.67	< .001*
**Female sex**	0.78	0.55–1.10	.159	0. 74	0.51–1.09	.132
**Married**	0.79	0.61–1.04	.090	1.05	0.79–1.41	.734
**Smoking status**^b^ (vs never)						
Current smoker	3.52	2.10–5.90	< .001*	2.60	1.47–4.61	.001*
Former smoker	2.39	1.46–3.92	< .001*	1.94	1.15–3.26	.013*
**Problem drinking**^c^	1.51	1.14–2.02	.005*	1.15	0.82–1.60	.412
**Body mass index**	0.95	0.93–0.98	< .001*	0.97	0.95–1.00	.053
**Depression**^d^	1.26	0.97–1.66	.088	1.14	0.86–1.51	.356
**Cancer site** (vs Other)						
Oral Cavity	0.93	0.62–1.41	.738	1.39	0.86–2.24	.179
Oropharynx	0.66	0.45–0.98	.037*	0.84	0.56–1.26	.402
Larynx	0.79	0.52–1.20	.264	0.85	0.54–1.34	.481
**Stage** (vs Stage I)						
Stage II	2.48	1.17–5.26	.018*	3.86	1.73–8.61	.001*
Stage III	2.42	1.19–4.94	.015*	3.98	1.82–8.71	.001*
Stage IV	2.90	1.53–5.49	.001*	4.54	2.20–9.39	< .001*
**ACE-27 comorbidity** (vs None)						
Mild	1.35	0.93–1.97	.113	1.14	0.77–1.68	.524
Moderate	2.11	1.43–3.11	< .001*	1.59	1.04–2.44	.031*
Severe	3.09	1.98–4.82	< .001*	2.09	1.30–3.36	.002*
**Surgery**	0.82	0.62–1.07	.135	0.79	0.58–1.09	.158
**Radiation treatment**	1.25	0.80–1.94	.322	0.74	0.43–1.28	.282
**Chemotherapy treatment**	1.33	1.98–1.81	.064	1.33	0.90–1.98	.152

^a^ Lowest quartile of income <$35,000.

^b^ Includes cigarettes, cigars, and pipe tobacco.

^c^ Alcohol Use Disorders Identification Test (AUDIT) ≥8.

^d^ Geriatric Depressive Scale Short Form ≥4.

*p < .05.

Multivariate analysis revealed that lowest quartile income (hazard ratio [HR], 1.5; 95% confidence interval [CI], 1.1–2.0), high school education or less (HR, 1.4; 95% CI, 1.1–1.9), and age in decades (HR, 1.4; 95% CI, 1.2–1.7) remained significant independent predictors of overall survival among head and neck cancer patients, while ethnicity/race, female sex, and marital status were not significant. Among covariates, smoking status, cancer stage, and comorbidities were significant, while problem drinking, BMI, cancer sites and treatment were no longer significant in the multivariate analysis.

Similar to the findings from overall survival, lower income, lower educational levels, black race, advanced age, being unmarried, current/former smoking, problem drinking, lower BMI, advanced stage, more severe comorbidity, and having chemotherapy were associated with worse cancer-specific survival in the univariate models (Table 4). In the multivariate model, a high school education or less (HR, 1.4; 95% CI, 1.0–2.1) and advanced age (HR, 1.3; 95% CI, 1.1–1.6) decreased cancer-specific survival rates (Table 4). Current/former smoking, lower levels of BMI, and advanced stage were significant covariates that predicted worse cancer-specific mortality, while comorbidity and chemotherapy were no longer significant.

**Table 4:** Univariate and multivariate Cox proportional hazards model for 5-year cancer-specific survival (N = 634 [147 events, 487 censored]).

	Univariate models			Multivariate models		
Parameter	Hazard ratio	95% CI	*p* value	Hazard ratio	95% CI	*p* value
**Low income**^a^	1.55	1.09–2.20	.014*	1.37	0.94–2.00	.103
**High school education or less**	1.69	1.22–2.35	.002*	1.44	1.01–2.06	.042*
**Ethnicity/race** (vs white)						
Black	1.76	1.03–3.00	.039*	1.26	0.75–2.46	.317
Hispanic, other (Native American)	1.05	0.43–2.58	.908	0.97	0.38–2.49	.954
**Age** (in decades)	1.25	1.07–1.45	.004*	1.33	1.11–1.60	.002*
**Female sex**	0.78	0.51–1.20	.261	0.72	0.46–1.14	.167
**Married**	0.72	0.52–0.99	.045*	0.92	0.65–1.31	.657
**Smoking status**^b^ (vs never)						
Current smoker	3.34	1.77–6.30	< .001*	2.37	1.18–4.75	.015*
Former smoker	2.52	1.38–4.60	.003*	2.12	1.13–3.99	.019*
**Problem drinking**^c^	1.45	1.02–2.05	.038*	1.00	0.67–1.50	.999
**BMI**	0.94	0.91–0.97	< .001*	0.96	0.93–0.99	.021*
**Depression**^d^	1.37	0.99–1.90	.060	1.21	0.87–1.70	.263
**Cancer site** (vs Other)						
Oral Cavity	1.03	0.62–1.71	.904	0.77	0.99–3.17	.056
Oropharynx	0.75	0.47–2.21	.236	0.93	0.57–1.53	.772
Larynx	0.78	0.46–1.33	.366	0.98	0.56–1.71	.935
**Stage** (vs Stage I)						
Stage II	3.55	1.14–11.00	.028*	5.39	1.66–17.54	.005*
Stage III	3.72	1.27–10.93	.017*	5.91	1.88–18.57	.002*
Stage IV	5.28	1.95–14.32	.001*	7.69	2.61–22.62	< .001*
**ACE-27 comorbidity** (vs None)						
Mild	1.06	0.70–1.61	.789	0.92	0.59–1.43	.710
Moderate	1.40	0.89–2.21	.150	1.12	0.68–1.85	.654
Severe	1.99	1.17–3.39	.012*	1.36	0.77–2.40	.296
**Surgery**	0.77	0.56–1.07	.120	0.71	0.48–1.05	.089
**Radiation treatment**	1.34	0.77–2.32	.302	0.62	0.32–1.21	.158
**Chemotherapy treatment**	1.61	1.09–2.37	.017*	1.55	0.94–2.55	.086

^a^ Lowest quartile of income <$35,000.

^b^ Includes cigarettes, cigars, and pipe tobacco.

^c^ Alcohol Use Disorders Identification Test (AUDIT) ≥8.

^d^ Geriatric Depressive Scale Short Form ≥4.

* *p* < .05.

With regard to disease-free survival, low income, low educational levels, and increased age were significantly associated with decreased disease-free survival in univariate models (Table 5). Ethnicity/race, sex, and marital status were not significant in relation to disease-free survival within 5 years post-diagnosis. In multivariate models, low income (HR, 1.4; 95% CI, 1.0–1.9), high school education or less (HR, 1.4; 95% CI, 1.1–1.9), and advanced age (HR, 1.2; 95% CI, 1.1–1.4) remained significant. Among covariates, former smoking at diagnosis, lower BMI, oral cavity cancer, advanced stage, and chemotherapy independently predicted lower disease-free survival.

**Table 5:** Univariate and multivariate Cox proportional hazards model for 5-year disease-free survival (N = 634 [207 events, 427 censored]).

	Univariate models			Multivariate models		
Parameter	Hazard ratio	95% CI	*p* value	Hazard ratio	95% CI	*p* value
**Low income**^a^	1.48	1.10–2.00	.009*	1.40	1.01–1.92	.042*
**High school education or less**	1.59	1.21–2.10	.001*	1.43	1.06–1.93	.018*
**Ethnicity/race** (vs white)						
Black	1.40	0.85–2.30	.186	1.10	0.64–1.90	.732
Hispanic, other (Native American)	1.16	0.54–2.46	.707	1.20	0.54–2.64	.654
**Age** (in decades)	1.20	1.06–1.36	.005*	1.23	1.06–1.44	.006*
**Female sex**	0.92	0.65–1.30	.639	0.93	0.64–1.34	.689
**Married**	0.85	0.65–1.13	.266	1.08	0.80–1.46	.601
**Smoking status**^b^ (vs never)						
Current smoker	2.46	1.46–4.12	.001*	1.69	0.96–3.00	.071
Former smoker	2.28	1.41–3.68	.001*	1.94	1.17–3.20	.010*
**Problem drinking**^c^	1.38	1.02–1.85	.035*	0.99	0.70–1.39	.956
**BMI**	0.94	0.91–0.97	< .001*	0.95	0.92–0.98	< .001*
**Depression**^d^	1.09	0.83–1.43	.545	1.02	0.77–1.36	.879
**Cancer site** (vs Other)						
Oral Cavity	1.19	0.77–1.84	.441	1.70	1.03–2.81	.038*
Oropharynx	0.85	0.56–1.29	.451	0.97	0.63–1.50	.893
Larynx	0.90	0.57–1.41	.638	1.01	0.62–1.64	.959
**Stage** (vs Stage I)						
Stage II	1.72	0.85–3.47	.134	1.89	0.89–4.03	.099
Stage III	1.68	0.87–3.24	.123	1.80	0.88–3.70	.109
Stage IV	2.08	1.18–3.68	.011*	2.01	1.04–3.87	.038*
**ACE-27 comorbidity** (vs None)						
Mild	1.01	0.71–1.43	.949	0.91	0.63–1.31	.598
Moderate	0.30	0.83–1.80	.302	1.05	0.69–1.60	.808
Severe	1.47	0.93–2.33	.103	1.15	0.70–1.89	.581
**Surgery**	1.04	0.79–1.37	.766	1.07	0.77–1.49	.704
**Radiation treatment**	1.48	0.92–2.37	.106	0.93	0.51–1.70	.821
**Chemotherapy treatment**	1.68	1.21–2.33	.002*	2.15	1.40–3.32	< .001*

^a^ Lowest quartile of income <$35,000.

^b^ Includes cigarettes, cigars, and pipe tobacco.

^c^ Alcohol Use Disorders Identification Test (AUDIT) ≥8.

^d^ Geriatric Depressive Scale Short Form ≥4.

*p < .05.

## Discussion

Controlling for other socioeconomic variables, education consistently predicted all survival outcomes (overall, cancer-specific, and disease-free survival) among head and neck cancer patients. Those with a high school education or less had a 41% higher hazard of dying from all causes, 44% higher hazard of dying from cancer-specific causes, and a 43% higher hazard of recurrence than the higher education reference group. This finding is consistent with the literature, which has found an inverse relationship between educational level and cancer survival.[51]

The reason for the association between education and outcomes is not clear. While it has been suggested that education may be a marker for poor health behaviors, such as smoking and drinking,[51] that was not the case in these analyses, since smoking, problem drinking, and nutritional status (BMI) were covariates. Education may also be related to income, which may influence treatment options; however, this was also included as a covariate. It may be because those with less education lack knowledge about the disease progress and early manifestations of cancer recurrence, which can lead to non-adherence to follow-up visits and cause higher recurrence rates among this population.

Median household income was another strong predictor of overall and disease-free survival in this study. Lower income status showed a 48% higher hazard of dying from all-cause and 40% higher hazard of recurrence than the higher income reference group. Previous studies[21, 52] proposed that poverty decreases survival through inadequate access to care and even though most of the patients in this study were insured and there was no discrepancy in stage at diagnosis among those who were of lower income, access to care is affected by other factors including resources and desire to receive follow up care. Unfortunately, we did not have information on compliance with follow-up appointments and related factors such as availability of tangible support (e.g., transportation) and desire to receive follow up visits.

Another explanation for the relationship between income and survival is that the stress associated with poverty (e.g., food insecurity) and undernutrition may suppress the immune system and induce inflammatory markers associated with survival.[33, 53, 54] In fact, Fig 1 shows that it is those with the lowest income (earning less than $35,000 per year) who are at greatest risk for poor survival. However, since we used census tract data as a proxy for individual income level, there may be residual confounding factors not properly controlled for in the analysis.

This population is particularly interesting to study in terms of disparities because, unlike other studies where income and education are highly correlated, thereby causing multicollinearity, there was not a strong association between the two in this study. One explanation is that many VA patients are lower income but have some college education from the GI Bill, and many blacks from Henry Ford Hospital in the Detroit area have less education but a fair income and health insurance related to auto-industry jobs. Hence, low education (controlling for income) and low income (controlling for education) were independent predictors of poor survival.

Both low income and low education were associated with black race. Black race was significantly associated with both decreased overall survival and cancer-specific survival in the univariate analysis, but was not significant in multivariate analysis, which controlled for other SES-related and covariates. Consistent with other studies,[12, 55] who found when blacks receive similar cancer treatment and medical care as whites, they tend to have similar disease outcomes.

Blacks did not present at a later cancer stage and were equally likely to receive radiation and surgery, but less likely to receive chemotherapy. This may be because of concerning comorbid conditions, functional status, patient biases, and institutional treatment differences at the time of the study and/or other variables that were not measured. Nonetheless, blacks did not have decreased survival as a result of receiving less chemotherapy.

Both education and income levels were significantly associated with cancer site, with higher education and higher income groups more likely to have cancers of the oropharynx. Since higher SES patients were less likely to smoke, they may have more non-smoking–related human papilloma virus (HPV)-positive cancers, which are more common in the oropharynx and have a more favorable prognosis.[9, 15, 56] Unfortunately, information about HPV was not available, so we were unable to control for HPV status in the analyses.

The IOM report, “Unequal Treatment,”[57] contains several recommendations on how to reduce disparities, which are related to the study findings, yet the IOM report focused primarily on race/SES disparities and did not address age, sex, and marital status. In this study, older patients were less likely to receive surgery and chemotherapy; it is unclear whether this is due to provider bias, patient preferences, or decisions made jointly based on comorbidities, which are higher in older persons. Older persons were also more likely to come from the VA Ann Arbor Healthcare System and Henry Ford Hospital, where chemotherapy was provided less frequently.

The survival curve for sex (Fig 1) is interesting in that the survival rate for both males and females was about the same for the first 2 years after diagnosis, but thereafter, females began to mimic the survival advantage shown in both general and head and neck cancer patient population trends.[17, 18, 58] While not significant, this survival advantage for females persisted even though females had less education, a lower likelihood of being married, and more depressive symptoms. However, females did have higher rates of surgery and lower rates of chemotherapy and radiation (all common treatment trends among those with a more favorable prognosis). It may also be related to the finding that women are more likely to have frequent clinic visits than men,[59] which may lead to early detection of the tumor.

Studies have shown that unmarried cancer patients are diagnosed at a later stage, are more likely to be untreated, and have a higher risk of dying.[35, 60] However, in this study, there was no relationship between marital status and cancer stage or treatment. Marriage significantly predicted better cancer-specific survival in univariate analysis, yet it was no longer significant in multivariate analysis. It is possible that low social support (not being married) or cultural attitudes (e.g., mistrust physicians) may have contributed to less frequent follow-up visits and suboptimal detection of recurrence, thus resulting in poorer survival. Special outreach may be required for those lacking support, such as those who are unmarried, widowed, or separated, to obtain needed care.

In terms of covariates, it was not surprising that about one-quarter of the patients smoked at diagnosis, and smoking was associated with all three survival outcomes, which has been documented in other studies.[29, 61, 62] While problem drinking has been shown to decrease survival in other studies of head and neck cancer patients[38, 62] and was a significant predictor of overall survival in univariate models, problem drinking was not significant in multivariate models. As demonstrated in other studies,[39, 63] higher BMI independently predicted better cancer-specific and disease-free survival. This finding supports the evidence that those with higher BMI better sustain the adverse effects of cancer treatments, thus leading to better survival outcomes.[39] Moreover, higher BMI may reflect better nutritional status, which may influence treatment options. Consistent with other studies,[19, 41, 64–66] those with oral cancer, advanced cancer stage, and more severe comorbidities had poorer survival. Chemotherapy was associated with poor survival as patients with more progressed stages generally receive chemotherapy. While treatment modality was evaluated in relation to survival, more detailed data on treatment modality (e.g., types and dosages of therapeutic regimens, treatment intervals) were not available to analyze. While a power analysis was not conducted a priori for this secondary data analysis, the odds ratios in the univariate results are very close to the ones in the multivariate results, which indicate that the adjustment effects of covariates are not significant. Moreover, the width of the confidence intervals in multivariate analyses was narrow enough to provide enough information to be confident about the finding, particularly those with narrow intervals that are far from 1.0.

## Conclusion

This longitudinal study of head and neck cancer patients, which controlled for a large number of confounders, showed that low income, low education, and advanced age predicted poor survival. This was true even though there was fairly equal access to care. Disparities in health habits, clinical characteristics, comorbidities, and treatment were also found among selected SES, age, sex, and marital status groups. Implementation of the recommendations of IOM report, “Unequal Treatment,” may reduce disparities among head and neck cancer patients.
